# Multiple Primary Cancers of the Colon, Rectum, and the Thyroid Gland

**DOI:** 10.4103/1319-3767.43277

**Published:** 2008-10

**Authors:** Ahmad Zubaidi

**Affiliations:** Department of Surgery, King Khalid University Hospital, Faculty of Medicine, King Saud University, Riyadh, Saudi Arabia

**Keywords:** Colon cancer, multiple primaries, neoplasms, prognosis, thyroid cancer

## Abstract

The major concern in the case of cancer, whether one or in worse case more than one, is the extent of treatment required and the prognosis. This article reports three cases with two cancers: colorectal cancer and thyroid cancer, in the same patient at the same time. It also discusses the related clinical presentation and management of the cancers, and a review of literature has been presented.

Colorectal cancer (CRC) is common in Saudi Arabia,[1] Matching with the reports in the developed countries,[2] CRC is ranked fourth among all cancers in the general Saudi population with an overall age-standardized rate (ASR) of 4.9/100 000 population.[4] It has a high propensity to metastasize; 30–40% of all patients have metastatic disease at the initial diagnosis.[5,6] Colorectal cancer is the third most common cancer in the world and the second leading cause of cancer-related deaths in the USA.[3] The most common sites of metastasis from CRC are the regional lymph nodes, the liver, the lung, and the peritoneum. CRC metastasis to the thyroid gland is rare, with only a few cases reported mainly in pathology-related literature, and a 4% incidence from autopsy data.[7]

In patients with known CRC, work-up often focuses on the primary disease, so that incidental coexistence of another primary malignant lesion can be missed. The definition of a second primary cancer here is a cancer that is detected in another organ of an individual patient, either synchronously or metachronously. Each tumor has to have a definite histological picture of malignancy, be distinctly separated, and clearly have no metastatic origin from the other tumor.

Generally, the prevalence of additional primary neoplasms in epithelial cancers is substantial. Dong ***et al.*** reported that 8.5% of 633 964 patients with known cancers were subsequently proven to have other and previously unrecognized types of second primary cancers.[8] Ueno ***et al.*** reported that 5.2% of 24 498 cancer patients had multiple, simultaneous, nonrelated cancers.[9] Sandeep ***et al.*** examined the incidence of a second primary cancer after a first primary thyroid cancer. They found that the risk of a second primary cancer after a first primary thyroid cancer was 1.31, with the following standardized incidence ratios of second primary cancers: colon (1.30) and rectum (1.23). This risk seems to increase with the duration of follow-up, with no substantial differences in risk being associated with age at the time of diagnosis of thyroid cancer, the sex of the patient, or the calendar period of diagnosis.[10]

On the other hand, neither the true incidence nor the prevalence of CRC with a second primary thyroid cancer is known. This article describes three cases of CRC simultaneously associated with thyroid cancer.

## CASE REPORTS

### Case 1

**Figure 1:**
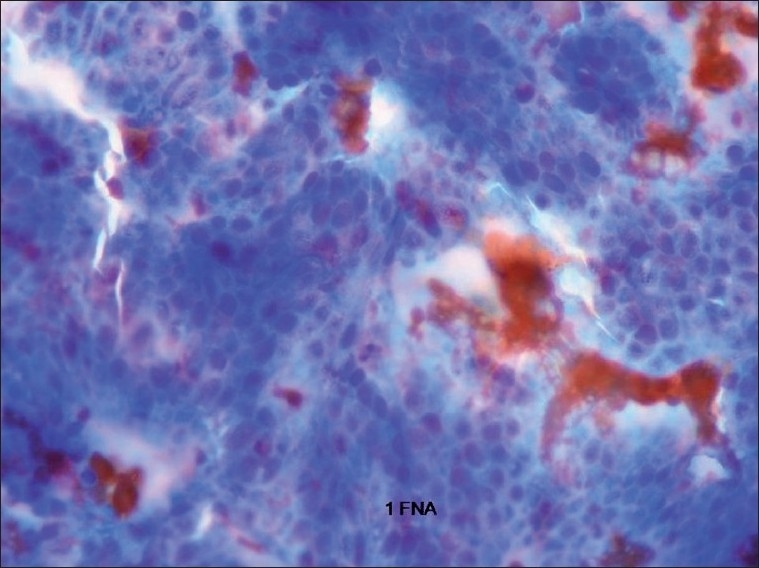
Fine needle aspiration of the thyroid lesions showed a picture confusing it with colon cancer

**Figure 2:**
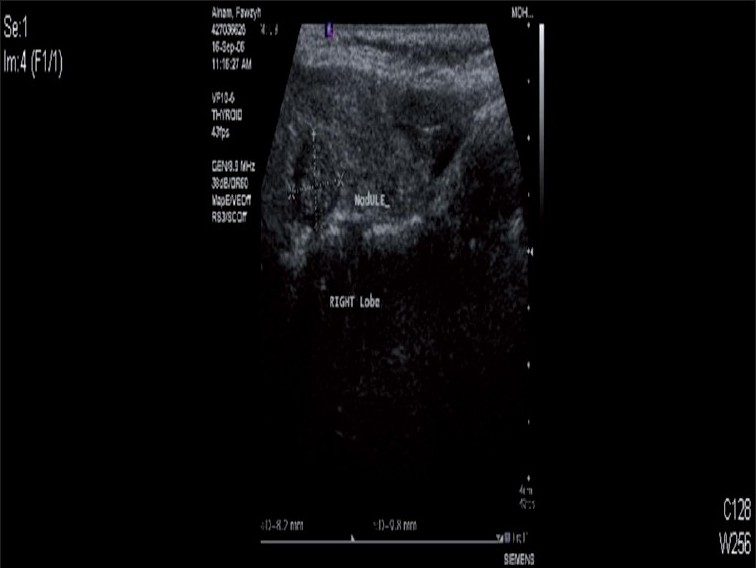
Ultrasound of the neck showing right thyroid lobe nodule

**Figure 3:**
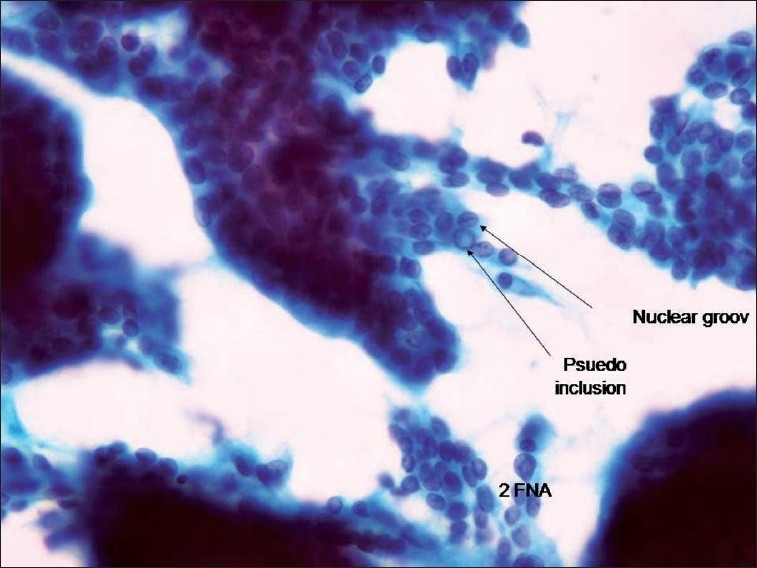
Repeated fine needle aspiration clearly showing features of papillary carcinoma of the thyroid

A 45-year-old female presented with three months' history of passing bright red blood per rectum, associated with tenesmus, but no history of abdominal pain, weight loss, or anorexia. She had a swelling on the right side of her neck for two years. No comorbid conditions were reported nor was there any family history of CRC. The physical examination showed a cervical lymphadenopathy on the right side of her neck. Chest and cardiovascular examination yielded normal results. Abdominal examination did not reveal any tenderness, organomegaly, or palpable masses. Digital rectal examination and rigid proctosigmoidoscopy revealed a rectal lesion that was 8 cm from the anal verge and was confirmed by colonoscopy. The rest of the colon was clear. Magnetic resonance imaging of the pelvis showed a rectal cancer confined to the wall. No evidence of any extrarectal involvement or regional lymphadenopathy was noted. Computer tomography (CT) of the abdomen and the chest did not reveal any metastasis. A rectal lesion biopsy showed the moderate development of adenocarcinoma. Unfortunately, transrectal ultrasonography was not performed because of the unavailability of the technology. Fine needle aspiration (FNA) of the cervical lymph node revealed a metastatic adenocarcinoma of colorectal origin [Figure 1]. The results of FNA were not convincing and were disregarded, especially as the lesion was more than two years old, with no other constitutional symptoms apart from the recent history of bleeding per rectum. A second physical examination of the neck was performed which revealed the same findings: cervical lymphadenopathy and no palpable thyroid mass. Neck ultrasonography was performed and confirmed a presence of nodularity in the right thyroid lobe [Figure 2]. FNA was performed again for both the thyroid nodule and the cervical lymphadenopathy. A second pathologist was asked to examine both FNA specimens and confirmed the diagnosis of a papillary carcinoma of the thyroid that had metastasized to the cervical lymph nodes [Figure 3]. The patient underwent open low anterior resection; pathology results were T2N0MX, moderate adenocarcinoma, and no lymphovascular invasion. Three weeks later, she underwent total thyroidectomy and a radical cervical lymphadenectomy. A final pathological examination confirmed the presence of a primary papillary carcinoma of the thyroid. Although no further adjuvant chemoradiotherapy was needed for the rectal cancer, the patient required radioactive iodine therapy for her thyroid disease.

### Case 2

A 36-year-old woman presented with a similar history that was about a year-old. There were no comorbid conditions or family history of CRC. Physical examination showed a right thyroid lobe swelling but no associated cervical lymphadenopathy. Abdominal examination did not reveal any tenderness, organomegaly, or palpable masses. Rigid proctosigmoidoscopy revealed a recto-sigmoid lesion that was 8 cm from the anal verge, but no synchronous lesion was detected by colonoscopy. CT scans of the abdomen and chest did not show any metastasis. Rectal lesion biopsy showed a moderately differentiated adenocarcinoma. Neck ultrasound revealed a multinodular goiter, while FNA of the thyroid showed a follicular tumor. The patient underwent a laparoscopic anterior resection. Pathology results were T3N1MX, a moderate adenocarcinoma, and no lymphovascular invasion. A month later, she underwent total thyroidectomy. A final pathological examination revealed a follicular carcinoma, and the patient was scheduled to receive adjuvant chemotherapy.

### Case 3

A 70-year-old man presented with a similar history that had been prevalent for several months, but there was no family history of CRC. Physical examination showed bilateral swelling of the thyroid lobes but no associated cervical lymphadenopathy. Abdominal examination did not reveal any tenderness, organomegaly, or palpable masses. Rigid proctosigmoidoscopy revealed a rectal lesion that was 10 cm from the anal verge although no synchronous lesion was detected using colonoscopy. CT scans of the abdomen and the chest were free of any metastasis. Biopsy of the rectal lesion showed a moderately differentiated adenocarcinoma. Ultrasonography of the neck revealed a multinodular goiter, while FNA of the thyroid showed a papillary carcinoma. Unfortunately, the patient refused all other medical interventions and was lost to follow-up.

## DISCUSSION

Technical advances in the diagnosis of cancer have increased the reported incidence of multiple primary cancers simultaneously diagnosed at the time of detection of CRC. Cancers develop because of accumulated mutations of multiple responsible genes, which increase the risk of developing cancers in other organs.[11–13] Therefore, the detection of a primary cancer should alert the treating team to exclude or effectively detect second cancers. Warren and Gates[14] defined multiple primary cancers by the following three conditions: (i) each tumor shows specific malignant findings; (ii) the two cancers should differ in site; and (iii) one tumor is not a metastatic focus from the other tumor. Most cases of second malignancies appear metachronously, with an overall prevalence of 17%.[15] Although a wide array of histological features has been observed with adenocarcinomas of the gastrointestinal tract, the precise pathogenic mechanism remains unclear. It is difficult to obtain data on second primary cancers in patients with colorectal cancers. Consequently, it is difficult to estimate the true incidence of CRC and primary thyroid cancer. However, occult primary thyroid carcinomas as well as metastases to the thyroid found at autopsy are not rare. Autopsy series have revealed incidental thyroid cancers in about 10% of primary thyroid lesions[16,17] and 1.25–24% of metastasis[18], but this is for all other cancers, including CRC.

Well-studied inherited mutations such as p53 (Li-Fraumeni syndrome),[19] mismatch-repair genes in Lynch syndromes,[20] and the adenomatous polyposis coli gene gene in familial adenomatous polyposis (FAP) patients predispose individuals and their families to a variety of multiple malignancies. Primary thyroid carcinoma occurs in 1–2% of all FAP patients and is mainly found in young women aged 30 years or below at the time of diagnosis. Moreover, histologically, 95% of these cases are the papillary type[21,22] and some show a peculiar cribriform pattern.[23]

The patients had never received any radiotherapy or chemotherapy for any previous disease; thus, iatrogenic carcinogenesis cannot explain the remarkable constellation of these neoplasms. These patients did not have a history of smoking, alcohol abuse, carcinogen exposure, or a personal or family history of cancer. However, no genetic testing has been offered to them because of the unavailability of these tests. These findings may be due to the evolution in clinical practice and/or changes in histological criteria; they may also be due to changing environmental and hormonal factors.

The presence of a thyroid nodule in a CRC (especially rectal cancer) patient presents a difficult diagnostic as well as management problem. Such a lesion could be benign, metastatic, or a new primary malignancy of the thyroid gland. In such cases, immunohistochemical staining for cytokeratins 7 (CK7) and 20 (CK20) is useful for differentiating primary thyroid cancers from metastatic adenocarcinomas of the colon and rectum. Thyroid carcinoma is usually positive for CK7 and negative for CK20, whereas colon cancer is negative for CK7 and positive for CK20.[24]

In patients with a malignancy, especially CRC, workup should include the ruling out of other lesions—metastatic cancers or other primary cancers, as well as FNA cytology of the suspected thyroid mass.[25] If an isolated, nonmetastatic, secondary thyroid cancer is found, depending on the nature of the disease, total thyroidectomy should be performed with or without lymph node dissection, with the assumption that it often prolongs disease-free survival and may occasionally be curative.[25–27]

## CONCLUSION

Clinical vigilance is required to reduce the rate of missing multiple primary cancers simultaneously present in the same patient. Such a discovery usually allows the clinician to correctly manage such a rare situation, and to improve the prognosis and the clinical judgment and subsequently adds to the accumulated knowledge.
